# A review on the role of LINC00467 in the carcinogenesis

**DOI:** 10.1186/s12935-022-02724-6

**Published:** 2022-10-13

**Authors:** Soudeh Ghafouri-Fard, Tayyebeh Khoshbakht, Bashdar Mahmud Hussen, Mohammad Taheri, Mohammadreza Hajiesmaeili

**Affiliations:** 1 Department of Medical Genetics, School of Medicine, Shahid Beheshti University of Medical Sciences, Tehran, Iran; 2 Phytochemistry Research Center, Shahid Beheshti University of Medical Sciences, Tehran, Iran; 3 Department of Pharmacognosy, College of Pharmacy, Hawler Medical University, Kurdistan Region, Erbil, Iraq; 4 Center of Research and Strategic Studies, Lebanese French University, Erbil, Kurdistan Region Iraq; 5 Institute of Human Genetics, Jena University Hospital, Jena, Germany; 6 Urology and Nephrology Research Center, Shahid Beheshti University of Medical Sciences, Tehran, Iran; 7 Critical Care Fellowship, Department of Anesthesiology, Loghman Hakim Hospital, Shahid Beheshti University of Medical Sciences, Tehran, Iran

**Keywords:** LINC00467, Cancer, lncRNA, Biomarker, Expression

## Abstract

LINC00467 is an example of long intergenic non-coding RNAs whose roles in human disorders are being identified. This gene coding LINC00467 is located on chromosome 1: 211,382,736 − 211,435,570 forward strand. This lncRNA has been firstly recognized through a microarray-based lncRNA profiling as an N-Myc target in neuroblastoma cells. Further studies have shown up-regulation of LINC00467 in different cancer including those originated from brain, gastrointestinal tract, lung and breast. It acts as a molecular sponge for miR-339, miR-138-5p, miR-107, miR-133b, miR-451a, miR-485-5p, miR-7-5p, miR-485-5p, miR-339-3p, miR-200a, miR-1285-3p, miR-299-5p, miR-509-3p, miR-18a-5p, miR-9-5p and miR-20b-5p. LINC00467 can regulate activity of NF-κB, STAT1, Wnt/b-catenin, Akt and ERK1/2 signaling pathways. Accumulating evidence indicates oncogenic role of LINC00467. The current review article aims at providing an overview of LINC00467 in the carcinogenesis.

## Introduction

Long non-coding RNAs (lncRNAs) are a group of transcripts having sizes larger than 200 nt. They are regarded as important epigenetic regulators that control epigenetic mechanisms principally in the nucleus, modulating transcription of genes through changing histone or DNA methylation and acetylation marks [1]. The majority of identified lncRNAs are transcribed by RNA polymerase II, thus having similar structures with mRNAs. While sharing many features with mRNAs, these widely expressed transcripts have distinct roles from mRNAs. Notably, function of lncRNAs is related with their particular subcellular localization [2]. In addition to modulation of chromatin function, lncRNAs can influence establishment and functions of nuclear bodies, change mRNAs stability and their translation and affect activity of signaling pathways [2].

GENECODE catalog of lncRNAs have classified these transcript into distinct categories of long intergenic non-coding (linc)-RNAs, antisense transcripts, intronic, and non-overlapping antisense transcripts [3]. LINC00467 is an example of the first group of lncRNAs whose roles in human disorders are being identified. This transcript is encoded by a gene located on chromosome 1: 211,382,736 − 211,435,570 forward strand. This gene has 28 transcripts with sizes ranging from 3536 bp (LINC00467-201) to 469 bp (LINC00467-204).

This lncRNA has been firstly recognized as an N-Myc target in neuroblastoma cells through a microarray-based transcriptome profiling [4]. Further studies have indicated abnormal expression of LINC00467 in a wide variety of cancer cell lines and clinical samples. Moreover, several studies have assessed functional roles of LINC00467 in xenograft models of cancers. The current review article aims at providing an overview of LINC00467 in the carcinogenesis through summarization of three mentioned lines of evidence.

## Cell line studies

### Sponging effects of LINC00467

Expression of LINC00467 has been shown to be elevated in acute myeloid leukemia (AML) cell lines. LINC00467 silencing has inhibited the malignant features of these cells. Notably, expression of miR-339 has been up-regulated after LINC00467 silencing. Moreover, expression of miR-339 target gene SKI has been decreased following this intervention. Since miR-339 silencing can chiefly eliminate the impact of LINC00467 silencing in AML cell lines, miR-339/SKI axis has been proposed as the molecular axis mediating the effects of LINC00467 [5].

In breast cancer cells, LINC00467 silencing has impeded proliferation, migratory potential, invasive features and epithelial-to-mesenchymal transition (EMT), while its up-regulation has led to opposite impacts. LINC00467 could down-regulate miR-138-5p through functioning as a molecular sponge for this miRNA. Moreover, LINC00467 could enhance expression of LIN28B through directly interacting with it [6]. Another *in silico* study in breast cancer has shown possible role of LINC00467 in the regulation of peroxisomal lipid metabolism and immune response through targeting miRNAs [7].

In cervical cancer cells, expression assays have detected high expression of LINC00467 and KIF23, and down-regulation of miR-107. LINC00467 has been shown to be mainly localized in the cytoplasm, where it acts as a molecular sponge for miR-107. LINC00467 silencing or miR-107 overexpression has blocked proliferation and decreased migration, invasion, and EMT [8].

In squamous cell carcinoma cells, LINC00467 can also enhance EMT through influencing activity of miR-299‐5p/USP48 axis [9].

Moreover, LINC00467 can influence response of hepatocellular cancer cells to Axitinib via acting as a molecular sponge for miR-509-3p and enhancing expression of PDGFRA [10]. miR-18a‐5p/NEDD9 [11] and miR-9-5p/PPARA [12] molecular axes are other routes of participation of LINC00467 in the pathoetiology of hepatocellular carcinoma as revealed through in vitro assays.

In osteosarcoma cells, LINC00467 has been shown to sponge miR‑217 and increase expression of KPNA4 [13] which facilitates progression of this type of cancer. Moreover, the sponging effect of LINC00467 on this miRNA leads to up-regulation of HMGA1 which enhances growth and metastatic abilities of these cells [14].

LINC00467 has also been shown to increase proliferation of lung adenocarcinoma cells through influencing miR-20b-5p/CCND1 activity [15]. Moreover, LINC00467 increases stemness of lung cancer cells through sequestering miR-4779 and miR‐7978 [16].

## Association of LINC00467 with transcription factors

Experiments in bladder cancer cells have shown the role of LINC00467 in enhancement of proliferation and invasive properties of these cells. Mechanistically, LINC00467 directly binds to NF-kb-p65 transcript, enhances its stability and promotes its nuclear translocation for further activation of the NF-κB signaling [17].

SiRNA-mediated LINC00467 silencing has suppressed proliferation, invasiveness and metastatic potential of colorectal cancer cells. Mechanistically, LINC00467 could affect expression of Cyclins D1 and A1, CDK2, CDK4, Twist1 and E‑cadherin [18].

LINC00467 can also promote invasive properties and block apoptosis of squamous cell carcinoma cells through sponging miR-1285-3p and enhancing expression of TFAP2A [19]. In hepatocellular carcinoma cells, LINC00467 has been shown to bind with IGF2BP3 and stabilize TRAF5, thus promoting proliferation and metastatic abilities of these cells [20].

## Upstream regulators of LINC00467

**Fig. 1:**
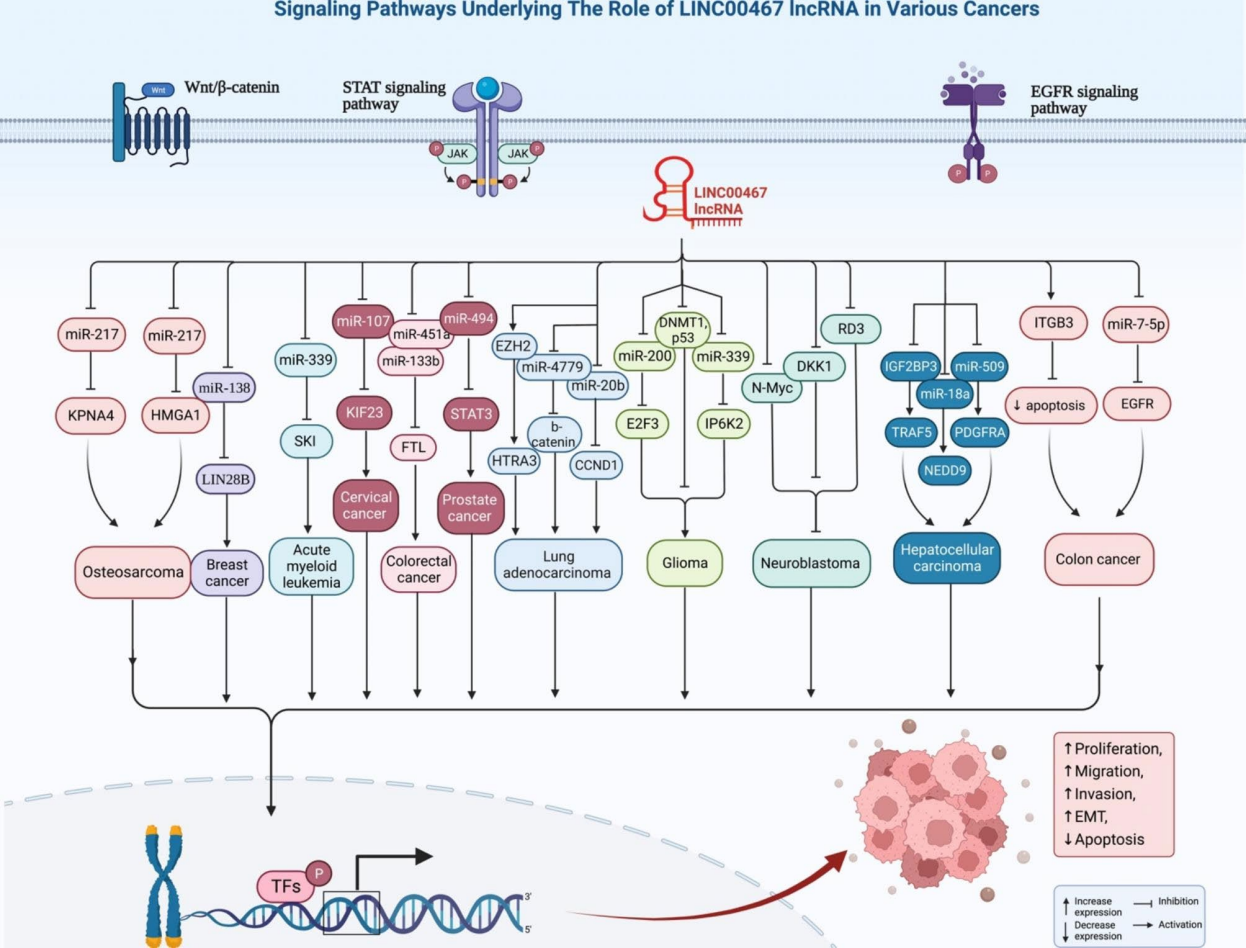
LINC00467 has oncogenic roles in several types of cancer, each with its own set of signaling pathways

Expression of LINC00467 has been shown to be suppressed by N-Myc. In fact, N-Myc directly binds to the promoter of LINC00467 gene, decreasing its promoter activity. N-Myc has also inhibited expression of the down-stream gene of LINC00467, i.e. RD3 via directly binding to its promoter (Fig. 1). SiRNA-mediated silencing of LINC00467 has led to up-regulation of the tumor suppressor gene DKK1. This intervention has also decreased viability of neuroblastoma cells and increased their apoptosis. Notably, co-transfection of LINC00467 siRNA and DKK1 siRNA has blocked the effect of LINC00467 silencing [4].

Table 1 shows function of LINC00467 in cell lines derived from different types of cancers.

**Table 1:** Function of LINC00467 in cell lines (∆: knock-down or deletion, EMT: epithelial-mesenchymal transition, DDP: cisplatin, 5-Fu: 5-fluor-ouracil)

Tumor type	Targets/ Regulators and Signaling Pathways	Cell line	Function	Reference
Acute myeloid leukemia	miR-339/SKI axis	HS-5, MV-4- 11, NB4, THP1, HL-60, and U937	∆ LINC00467: ↓ proliferation, migration, invasion, ↑ apoptosis and cell cycle arrest	[5]
Bladder cancer	NF-κB signaling pathway	T24 and RT4	↑↑ LINC00467: ↑ proliferation and invasion via binding to NF-kb-p65 mRNA to stabilize its expression and binding to NF-kb-p65 to promote its translocation into the nucleus to activate the NF-κB signaling pathway	[17]
Breast cancer	miR-138-5p and LIN28B	SKBR-3, MCF-7, T47D, MDA-MB-231, and BT-549	∆ LINC00467: ↓ proliferation, migration, invasion and EMT process via interacting with miR-138-5p and LIN28B directly	[6]
		MCF-7 and MDA-MB-231	LINC00467, regulated by Copy Number Amplification and DNA demethylation, is involved in oxidative lipid metabolism and immune infiltration in Breast Cancer.	[7]
Cervical cancer	miR-107/KIF23 axis	HeLa (CL-0101) and SiHa (CL-0210)	∆ LINC00467: ↓ proliferation, migration, invasion and EMT process	[8]
Colorectal cancer		NCM460, HT29, SW480, SW620 and HCT116	∆ LINC00467: ↓ proliferation, invasion, metastasis and EMT process	[18]
	miR-133b/FTL axis	SW480, Caco2, SW620, HT29, HCT116 and HIEC	↑↑ FTL (which regulates via LINC00467): ↑ resistance to 5-FU treatment and metastasis	[21]
	miR-451a	HCT116, HT29 and SW620 and NCM460	∆ LINC00467: ↓ proliferation, ↑ apoptosis	[22]
Esophageal carcinoma	miR-485-5p/DPAGT1 axis	KYSE510, TE-5, TE‐7, KYSE‐200 and Het‐1 A	↑↑ LINC00467: ↑ proliferation, and ↓ apoptosis	[23]
Gastric cancer	miR-7-5p/EGFR axis	MKN45, HGC-27, NCI-N87, AGS, MKN28 and GES-1	∆ LINC00467: ↓ proliferation, migration, invasion	[24]
	ITGB3		↑↑ LINC00467: ↑ viability, proliferation and ↓ apoptosis via increasing ITGB3 level	[25]
Glioma	miR-485-5p	NHA, LN299, A172, U87, and U251	∆ LINC00467: ↓ proliferation, invasion and ↑ apoptosis	[26]
	miR-339-3p/IP6K2 axis	HEB, LN229, LN308, U87, and U251	∆ IP6K2 (which regulates by LINC00467): ↓ proliferation, migration, invasion	[27]
	DNMT1, p53	LN229, LN308, U87, LN229 and HEB	↑↑ LINC00467: ↑ proliferation, invasion and cell cycle progression via inhibition of p53 expression by binding to DNMT1	[28]
	miR-200a/E2F3 axis	U87, U251, SHG-44, U-118 MG and HA	∆ LINC00467: ↓ proliferation, viability, migration, invasion and ↑ apoptosis	[29]
	miR-339-3p/IP6K2 axis	U87, U251, A172, U373 and NHA	∆ LINC00467: ↓ proliferation and ↑ apoptosis	[30]
Head and neck squamous cell carcinoma	miR-1285-3p/TFAP2A axis	HN4, HN6, SCC-4, SCC-9 and HOK	∆ LINC00467: ↓ invasion and ↑ apoptosis	[19]
	miR-299-5p/USP48 axis	HN6, SCC25, HN4, Cal27 and SCC4 and HOK	∆ LINC00467: ↓ cell growth, migration and EMT process	[9]
Hepatocellular carcinoma	IGF2BP3 and TRAF5	THLE-3 and HCCLM3, Hep3B, HepG2 and Huh‐7	∆ LINC00467: ↓ proliferation, metastasis, and ↑ apoptosis LINC00467 via binding with IGF2BP3 to increase the mRNA stability of TRAF5 in HCC induces cell proliferation and metastasis.	[20]
	miR-509-3p/PDGFRA axis	L02, MHCC97H, Hep3B, HepG2, Huh7, and HCCLM3	∆ LINC00467: ↓ proliferation, invasion, ↑ apoptosis and cellular sensitivity to Axitinib	[10]
	NR4A3	QSG-7701 and HCC cell lines HepG2, SK‐HEP‐1 and Huh7	↑↑ LINC00467: ↑ proliferation and migration LINC00467 through Dicer-dependent RNA splicing inhibited NR4A3 expression in post‐transcriptional level.	[31]
	miR-18a-5p/NEDD9 axis	Bel-7402, SMMC‐7721, HepG2, Hep3B, HCCLM3, and LO2	∆ LINC00467: ↓ growth, motility and ↑ apoptosis	[11]
	miR-9-5p/PPARA axis	SMMC-7721 and HepG2	↑↑ LINC00467: ↓ viability, proliferation, migration and invasion	[12]
Lung adenocarcinoma	miR-20b-5p/CCND1 axis	H1299, H23, A549, HCC827 and IMR90	∆ LINC00467: ↓ proliferation and ↑ cell cycle arrest	[15]
	miR-4779 and miR-7978	SPC-A1, A549, Calu3, and H1299, BEAS‐2B	∆ LINC00467: ↓ proliferation, stemness and ↑ apoptosis	[16]
	STAT1, DKK1/Wnt/b-catenin signaling pathway	H1299, Calu, SPC- A1, and A549, BEAS- 2B	↑↑ LINC00467: ↑ proliferation and migration STAT1 increased LINC00467 expression by acting as a transcription activator. LINC00467 is involved in epigenetically silencing DKK1 to activate Wnt/β-catenin signaling pathway.	[32]
	EZH2 and HTRA3	H1299, A549, PC9 and 16HBE	↑↑ LINC00467: ↑ proliferation, migration and invasion, and ↓ apoptosis Via recruiting EZH2 to the HTRA3 promoter to inhibit its expression	[33]
Neuroblastoma	N-Myc, RD3, DKK1	BE(2)-C and Kelly	∆ LINC00467: ↓ viability, reduction in RD3 mRNA expression, thus reduces cell survival by inducing DKK1 expression, ↑ apoptosis N-Myc inhibits linc00467 expression by direct binding to its gene promote.	[4]
Non-small cell lung cancer	Akt signaling pathway	H1299 and A549	∆ LINC00467: ↓ cell growth and metastasis via regulating the Akt signaling pathway	[34]
	miR-125a-3p/SIRT6 axis and ERK1/2 signaling pathway	A549 and H1299	∆ LINC00467: ↓ malignancy and DDP resistance via inhibiting SIRT6 and inactivating the ERK1/2 signaling pathway	[35]
Osteosarcoma	miR-217/KPNA4 axis	Hfob1.19, Saos2, MG63, U2OS and HOS	∆ LINC00467: ↓ proliferation, migration, invasion and EMT process	[13]
	miR-217/HMGA1 axis	HOS, MG63, Saos2 and SJSA1 and Hfob1.19	∆ LINC00467: ↓ proliferation, migration, invasion and EMT process and ↑ apoptosis	[14]
Prostate cancer	miR-494-3p/STAT3 axis	VCaP, LNCaP, 22RV1, PC3, DU145, HrPEC and RWPE-1	∆ LINC00467: ↓ cell growth, cell cycle progression, migration, and invasion and also ↓ cell migration via M2 macrophage polarization	[36]
Testicular germ cell tumor		NCCIT and Tcam-2	∆ LINC00467: ↓ migration, invasion, and clone formation	[37]

## Mouse studies

Up-regulation of LINC00467 has enhanced breast cancer growth, whereas its silencing has inhibited lung metastases in vivo [6]. Furthermore, LINC00467 knock down or miR-107 over-expression has suppressed tumorigenic ability of cervical cancer cell in xenograft models [8]. Similar studies in AML, bladder cancer, colorectal cancer, esophageal carcinoma, glioma, hepatocellular carcinoma, lung cancer and prostate cancer have consistently confirmed oncogenic effects of LINC00467 (Table 2).

**Table 2:** Function of LINC00467 in animal models. (∆: knock-down or deletion, NOD-SCID: immunodeficient, AML: Acute myeloid leukemia)

Tumor type	Animal models	Results	Reference
Acute myeloid leukemia	NOD-SCID mice	∆ LINC00467: ↓ AML progression in immunodeficient mice	[5]
Bladder cancer	5-week-old female nude mice	∆ LINC00467: ↓ proliferation and tumor formation	[17]
Breast cancer	5-week-old female Balb/c nude mice	∆ LINC00467: ↓ tumor growth and metastasis	[6]
Cervical cancer	40 5-week-old male BALB/c nude mice	∆ LINC00467: ↓ tumor volume and weight	[8]
Colorectal cancer	4-week-old male Balb/c nude mice	∆ FTL (which regulates via LINC00467): ↓ metastasis	[21]
Esophageal carcinoma	4–6 week-old female BALB/c nude mice	∆ LINC00467: ↓ tumor growth, volume, weight and size	[23]
Glioma	4-week-old BALB/c-nude mice	↑↑ IP6K2 (which is regulated by LINC00467): ↑ tumor volume and weight	[27]
	Male athymic BALB/c nude mice	∆ LINC00467: ↓ tumor volume and weight	[29]
Hepatocellular carcinoma	Male BALB/c nude mice	∆ LINC00467: ↓ tumor growth	[31]
Lung adenocarcinoma	6-week-old female nude mice	∆ LINC00467: ↓ tumor volume and weight	[15]
Non-small cell lung cancer	8-week-old male BALB/c nude mice	∆ LINC00467: ↓ tumor growth	[34]
Prostate cancer	6-week-old male BALB/c nude mice	∆ LINC00467: ↓ tumor growth, volume and weight	[36]

## Clinical studies

Assessment of expression data from a GEO dataset and the TCGA database has revealed up-regulation of LINC00467 in bladder cancer samples and negative correlation between its expression and patients’ prognosis [17]. Expression assays in patients with breast cancer has also verified over-expression of LINC00467 in cancerous tissues compared with nearby normal samples. Moreover, up-regulation of LINC00467 has been associated with poor overall survival (OS) [6]. Another study has indicated association between LINC00467 over-expression and tumor metastases and poor prognosis. Genomic and epigenetic analyses have shown the impact of copy number amplification, chromatin configuration, and methylation status of DNA on expression of this lncRNA. Copy number amplification and up-regulation of LINC00467 has been associated with the lower levels CD8 + and CD4 + T cells infiltrations [7]. LINC00467 level has also been reported to be elevated in colorectal cancer tissues compared with normal colon mucosal counterparts. *In silico* analyses available datasets have confirmed correlation between over-expression of LINC00467 and poor OS and recurrent-free survival rate [18]. The association between over-expression of LINC00467 and poor clinical outcome has been verified in different cancers, including bladder cancer, breast cancer, colorectal cancer, glioma, lung cancer, osteosarcoma and testicular germ cell tumor (Table 3).

**Table 3:** Dysregulation of LINC00467 in clinical samples (ANCTs: adjacent non-cancerous tissues, OS: Overall survival, DFS: disease-free survival, AML: Acute myeloid leukemia, GEPIA: Gene Expression Profiling Interactive Analysis, GEO: Gene Expression Omnibus, RFS: recurrent-free survival)

Tumor/disease type	Samples	Expression (Tumor vs. Normal)	Kaplan-Meier analysis (impact of LINC00467 up-regulation)	Association of high expression LINC00467 with clinicopathologic characteristics	Association studies	Reference
Acute myeloid leukemia	34 AML patients and 40 healthy controls	Upregulated				[5]
Bladder cancer	GEO (GSE133624 n = 55) and TANRIC (n = 271) database 6 pairs of tumor tissues and ANCTs	Upregulated	Shorter DFS			[17]
Breast cancer	TCGA datasets: 1,091 tumor tissues and 113 normal tissues 70 pairs of tumor tissues and ANCTs	Upregulated	Shorter OS	Tumor stage and lymph node metastasis		[6]
	GEO database: GSE7904, GSE45827, GSE65194, GSE22820 and GSE38959	Upregulated				[7]
Cervical cancer	GEO database: (GSE7803, GSE9750, and GSE63514) 54 pairs of tumor tissues and ANCTs	Upregulated		Tumor size, differentiation, and tumor-node-metastasis stage		[8]
Colorectal cancer	GEO (GSE22598, GSE37364, and GSE50760) and GEPIA databases 45 pairs of tumor tissues and ANCTs	Upregulated	Shorter OS and RFS			[18]
	20 patients and 20 healthy controls	Upregulated in FTL (which regulates via LINC00467)				[21]
	31 pairs of tumor tissues and ANCTs	Upregulated		TNM stage and serum CEA level		[22]
Esophageal carcinoma	GEPIA database: 182 tumor tissues and 286 normal tissues 44 pairs of tumor tissues and ANCTs	Upregulated		Tumor size, lymph node metastasis, advanced TNM stage		[23]
Gastric cancer	TCGA data (211 pairs of tumor tissues and ANCTs)	Upregulated				[24]
Glioma	30 pairs of tumor tissues and ANCTs	Upregulated	Shorter OS	Tumor grade		[26]
	30 pairs of tumor tissues and ANCTs	Upregulated in IP6K2 (which regulated by LINC00467)	Shorter OS	Advanced stages and metastatic glioma		[27]
	TCGA STAD database (glioma tissues (n = 163) and normal tissues (n = 207))	Upregulated				[28]
	GEPIA database (glioma tissues (n = 163) and normal tissues (n = 207)) 30 pairs of tumor tissues and ANCTs	Upregulated				[30]
Head and neck squamous cell carcinoma	35 pairs of tumor tissues and ANCTs	Upregulated				[19]
Hepatocellular carcinoma	GSE6764: 35 tumor tissues and 40 non-cancerous liver tissues 56 pairs of tumor tissues and ANCTs	Upregulated		Tumor size and vascular invasion		[31]
	GEPIA database: 369 tumor tissues and 160 normal tissues 20 pairs of tumor tissues and ANCTs	Upregulated				[11]
	65 pairs of tumor tissues and ANCTs	Downregulated				[12]
Lung adenocarcinoma	33 pairs of tumor tissues and ANCTs	Upregulated	Shorter OS			[15]
	38 pairs of tumor tissues and ANCTs	Upregulated				[16]
	GEO (GSE19804, GSE19188, GSE30219, GSE27262 data set) and TCGA TANRIC databases 35 pairs of tumor tissues and ANCTs	Upregulated		Larger tumor sizes and later TNM stages		[33]
Non-small cell lung cancer	GEO (GSE33532), GEPIA databases	Upregulated	Shorter OS and DFS	Advanced clinical stages and poor outcome		[34]
Osteosarcoma	36 pairs of tumor tissues and ANCTs	Upregulated	Shorter OS			[13]
	44 pairs of tumor tissues and ANCTs	Upregulated	Shorter OS	Tumor size, TNM stage, Distant metastasis		[14]
Prostate cancer	the GTEx and TCGA databases (49 pairs of tumor tissues) 22 pairs of tumor tissues and ANCTs	Upregulated				[36]
Testicular germ cell tumor	14 tumor tissues and 9 normal tissues	Upregulated	Shorter OS and DFS	Tumor stage		[37]

## Discussion

Numerous studies have indicated up-regulation of LINC00467 in different types of cancers. Mechanistically, this lncRNA can be up-regulated through DNA demethylation and copy number variations.

The sponging effect of LINC00467 on miRNAs has been well assessed in different cancer cell lines. Through this mechanistical route, LINC00467 can affect activity of miR-339/SKI, miR-107/KIF23, miR-133b/FTL, miR-485-5p/DPAGT1, miR-7-5p/EGFR, miR-339-3p/IP6K2, miR-200a/E2F3, miR-1285-3p/TFAP2A, miR-299-5p/USP48, miR-509-3p/PDGFRA, miR-18a-5p/NEDD9, miR-9-5p/PPARA, miR-20b-5p/CCND1, miR-125a-3p/SIRT6, miR-217/KPNA4, miR-217/HMGA1 and miR-494-3p/STAT3 axes. Moreover, LINC00467 can influence activity of NF-κB, STAT1, Wnt/b-catenin, Akt and ERK1/2 signaling pathways. Most notably, LINC00467 has been shown to increase EMT in breast, cervical, colorectal, head and neck and prostate cancer as well as osteosarcoma. Thus, strategies to decrease expression of LINC00467 are expected to affect tumor invasion and metastasis.

LINC00467 has a possible role in the tumor microenvironment and immune evasion. Copy number variations within LINC00467 have been associated expression levels of this lncRNA, immune infiltration in lung adenocarcinoma and poor clinical outcome [38]. Moreover, LINC00467 expression in breast cancer has been associated with immune infiltration [7].

Up-regulation of LINC00467 has been associated with poor prognosis of patients with bladder cancer, breast cancer, colorectal cancer, glioma, lung cancer, osteosarcoma and testicular germ cell tumor. Thus, LINC00467 is a putative prognostic marker in cancers. However, the potential of this lncRNA as a diagnostic marker has not well studied. Future studies should focus on this aspect. Expression assays of LINC00467 particularly in biofluids such as serum and urine would pave the way for establishment of non-invasive methods for cancer diagnosis.

Identification of additional miRNA targets of LINC00467 is expected to clarify the molecular mechanisms and signaling pathways being affected by this lncRNA. This would help in design of novel and efficient targeted therapies for cancer. Based on the critical roles of LINC00467 in the regulation of cell apoptosis, it is expected that modification of its expression affects response of cancer cells to anti-cancer modalities. This function of LINC00467 has been verified in hepatocellular carcinoma cells where its silencing has enhanced sensitivity to Axitinib ([10].

Finally, the presence of single nucleotide polymorphisms within LINC00467 would affect expression or function of this lncRNA. Therefore, genotyping of these variants would help in recognition of risk factors for different types of cancer.

## Conclusions and future prospects

LINC00467 is regarded as an oncogenic lncRNA in humans. Thus, strategies to down-regulate its expression are theoretically effective in reduction of tumor burden. The most challenging issue in this regard is establishment of effective ways to convey LINC00467-targetted therapies in a specific way to cancer cells and avoid off-target effects.

## Data Availability

The analyzed data sets generated during the study are available from the corresponding author on reasonable request.
